# Incorporating a Shortened Long-Term Effectiveness End Point Into Catheter Ablation Trials: Statistical Properties and Implications

**DOI:** 10.1161/CIRCEP.125.014773

**Published:** 2026-09-18

**Authors:** Jason G. Andrade, Stephen Hynes, Farid Jamshidian, Andrea Natale

**Affiliations:** 1 Department of Medicine, Vancouver General Hospital, Vancouver, Canada (J.G.A.).; 2 Center for Cardiovascular Innovation, Vancouver, Canada (J.G.A.).; 3 Department of Medicine, Montréal Heart Institute, Université de Montréal, Montréal, Canada (J.G.A.).; 4 Biosense Webster, Inc., part of Johnson & Johnson MedTech, Irvine, CA (S.H., F.J.).; 5 Texas Cardiac Arrhythmia Research Foundation, Austin, TX (A.N.).; 6 Division of Cardiology, Department of Biomedicine and Prevention, University of Tor Vergata, Rome, Italy (A.N.).

**Keywords:** atrial fibrillation, atrial flutter, catheter ablation, clinical trial, survival rate

## Abstract

**BACKGROUND:**

Evidence regarding the use of a 6-month effectiveness end point for trials of atrial fibrillation ablation is limited. This study aims to evaluate the relationship between ablation effectiveness end point detection and follow-up duration, with the secondary goal of informing the design of clinical trials of paroxysmal atrial fibrillation ablation.

**METHODS:**

Patient-level data from 4 prospective studies were aggregated. Long-term effectiveness end points were calculated with (1) a 3-month blanking period and 12-month follow-up (current) and (2) a 2-month blanking period and 6-month follow-up (proposed). Sensitivity, specificity, and agreement were calculated for each study under the current and proposed end points. A linear approximation method was used to extrapolate the 6-month survival estimate to 12 months, and the predicted 12-month survival rates were compared with the actual 12-month survival rates. Using the linear approximation, a new performance goal was derived and compared against the 6-month survival of each study.

**RESULTS:**

The comparative analysis between the current and proposed end points demonstrated concordances ranging from 83% to 92% and sensitivities ranging from 57% to 76% across the 4 recent clinical trials. The linear approximation method produced an estimated 12-month survival rate of the 4 pooled studies of 72.5%, compared with the true 12-month survival rate of 71.0% (−1.5% error). When compared against the true 12-month survival rate in each study individually, the estimated 12-month survival rate resulted in errors ranging from −3.6% to 0.3%. A new performance goal of 56.2% is proposed for trials using a 6-month end point.

**CONCLUSIONS:**

The proposed end point of a 6-month follow-up duration, inclusive of a 2-month blanking period, demonstrated reasonable levels of agreement with the current 12-month effectiveness end point. Enabling historical comparison, a 12-month survival rate can be reliably extrapolated from a 6-month survival rate using linear approximation.

**WHAT IS KNOWN?**

- Most arrhythmia recurrences are detected by month 6 postprocedure.
- Patients with recurrence in the first month after the blanking period have a higher atrial fibrillation burden than those with recurrence >1 month after the blanking period.

**WHAT THE STUDY ADDS**

- The current study demonstrates that a 6-month follow-up duration, inclusive of a 2-month blanking period, has reasonable agreement with the usual 12-month effectiveness end point.
- Success at 12 months can be accurately extrapolated from a 6-month follow-up duration, enabling comparison with historical studies.
- A performance goal of 56.2% freedom from recurrent atrial tachyarrhythmia is proposed for single-arm studies using a 6-month end point.

Clinical research studies evaluating atrial fibrillation (AF) interventions are costly, complex, and logistically challenging. Expert consensus has recommended that studies evaluating catheter ablation for AF employ a minimum duration of follow-up of 12 months, inclusive of a 3-month blanking period (ie, 9 months of active arrhythmia monitoring).^1^ Recently, the recommended blanking period duration was shortened from 3 to 2 months, while the total follow-up duration was maintained at 12 months, effectively lengthening the period of active follow-up from 9 to 10 months.^2^ Although employed widely, the empirical 12-month follow-up duration lacks validation.

Given the time and resources required for clinical studies and randomized trials, even a small change in the minimum duration of postablation follow-up can have major consequences. For example, reducing the total follow-up duration from 12 to 6 months would decrease the demands on patients, accelerate data generation, facilitate earlier dissemination of results to practice, and result in substantial operational savings (eg, lower study costs), democratizing clinical research. In the case of novel pharmacological and nonpharmacological therapeutics, a reduction in follow-up duration could enable earlier regulatory approval and adoption into practice.

The goal of the current study was to evaluate the relationship between ablation effectiveness end point detection and follow-up duration, with the secondary goal of informing the design of clinical trials of paroxysmal AF ablation.

## Methods

We performed a patient-level analysis of 4 multicenter prospective clinical studies: AdmIRE (Assessment of Safety and Effectiveness in Treatment Management of Atrial Fibrillation With the BWI IRE Ablation System; URL: https://www.clinicaltrials.gov; Unique identifier: NCT05293639), Q-FFICIENCY (Evaluation of QDOT MICRO Catheter for Pulmonary Vein Isolation in Subjects With Paroxysmal Atrial Fibrillation; URL: https://www.clinicaltrials.gov; Unique identifier: NCT03775512), STELLAR (Safety and Effectiveness Evaluation of the Multi-Electrode Radiofrequency Balloon Catheter for the Treatment of Symptomatic Paroxysmal Atrial Fibrillation; URL: https://www.clinicaltrials.gov; Unique identifier: NCT03683030), and SURPOINT (Evaluation of VISITAG SURPOINT Module With External Processing Unit; URL: https://www.clinicaltrials.gov; Unique identifier: NCT03624881).^3–6^ These 4 trials included populations of patients with antiarrhythmic drug–refractory paroxysmal AF and employed comparable noninvasive arrhythmia monitoring protocols (Table 1). For each study, the primary effectiveness end point was the time-to-first occurrence of any atrial tachyarrhythmia (AF, atrial tachycardia [AT], or atrial flutter [AFL]) at 1 year of follow-up, inclusive of a 3-month blanking period (ie, 9 months of active arrhythmia monitoring). All patients provided informed consent.

**Table 1.**
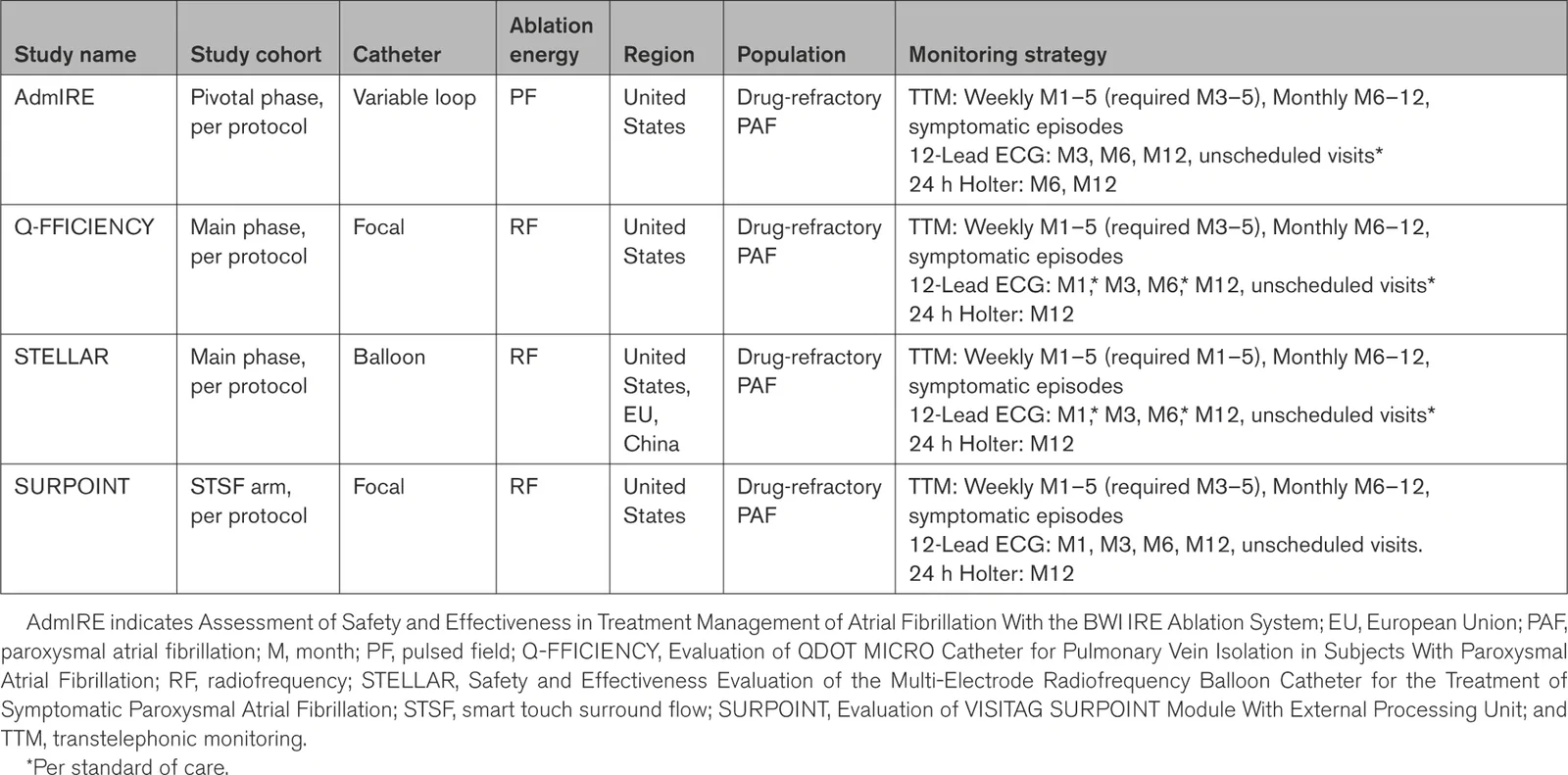
Monitoring Strategies Used in the Clinical Studies

Requests for access to the study data can be submitted through the Yale Open Data Access Project site at http://yoda.yale.edu. Johnson & Johnson MedTech has an agreement with the Yale Open Data Access Project to serve as the independent review panel for the evaluation of requests for clinical study reports and patient-level data from investigators and physicians for scientific research that will advance medical knowledge and public health.

### Effectiveness End Point

Clinical effectiveness was defined as freedom from a failure event, which was defined as any of the following:

1. AF/AT/AFL recurrence during the evaluation period
2. New antiarrhythmic drug usage during the evaluation period
3. Escalation in dosage of a currently employed antiarrhythmic drug during the evaluation period
4. More than 1 repeat ablation during the blanking period
5. Any repeat ablation during the evaluation period
6. Direct cardioversion for AF/AT/AFL during the evaluation period.

The current 12-month effectiveness end point analysis employed a 3-month blanking period (0–90 days) and defined the evaluation period as postablation days 91 to 365.^1^

The proposed 6-month effectiveness end point employed a 2-month blanking period (0–60 days) and defined the evaluation period as postablation days 61 to 180. The 2-month blanking period was selected in concordance with the updated 2024 consensus statement.^2^

### End Point Concordance

For each of the included studies, 2-by-2 tables were created, counting the number of successes and failures under the current and proposed end points. Concordance, sensitivity, specificity, positive predictive value, and negative predictive value were calculated for each study with 95% exact CIs, using the current effectiveness parameters as ground truth.

### Linear Approximation Prediction

We hypothesized that, after a certain time point, the recurrence rate would remain both low and relatively constant over time. As a result, the survival function could be reasonably approximated by a linear function between months 6 and 12. To estimate this constant hazard rate, we used data from postablation months 5 to 6 and extrapolated that to the interval between months 6 and 12, operating under the assumption that the hazard rate had stabilized by month 5. The specific window between months 5 and 6 was considered since it was sufficiently long enough in duration to obtain estimates of the hazard with reasonably low variance (Table S1), as well as to reflect clinical trial practice, where study participants are expected to have their 6-month follow-up visit between months 5 and 7 postprocedure.

Combining data from all 4 studies, a Kaplan-Meier survival curve using all long-term effectiveness failures from months 2 through 12 was created, assuming a 2-month blanking period, and the 5-month, 6-month, and 12-month survival probabilities were estimated. Then, we calculated a linear approximation using the following formula:


$$Linear\;Approximation=\frac{5m\;Survival-6m\;Survival}{\left(52/12\right)}$$

This formula corresponds to the average weekly change in survival rate, estimated based on the window between months 5 and 6. This estimated rate was used to extrapolate from the 6-month rate to the 12-month rate. The predicted 12-month survival was estimated with the following formula:


$$\begin{array}{c} 12m\;predicted=\left(6m\;survival-[26\times linear\;approximation]\right) \end{array}$$

Additionally, the predictive accuracy of 12-month survival, based on 6-month survival, was estimated and reported for the pooled cohort and each individual study using the following formula:


$$\begin{array}{c} Error=12m\;survival-12m\;predicted \end{array}$$

### Sensitivity Analyses

Three sensitivity analyses were performed to validate the approach and examine whether the accuracy of the approach holds under slight variations in data and scenarios. These sensitivity analyses included the following: (1) an examination of each individual study, (2) a pooled analysis of all studies but employing a 3-month blanking period, and (3) an alternative long-term effectiveness end point, using 3 failure modes ([1] AF/AT/AFL recurrence during the evaluation period, [2] any repeat ablation during the evaluation period, and [3] direct cardioversion for AF/AT/AFL during the evaluation period).

### Calculating a New PG

Single-arm trials currently use a performance goal (PG) of 50% at 12 months of follow-up.^1,2^ Given the proposed truncation of follow-up, an updated PG was derived using the estimated pooled slope from the linear approximation using the following formula:

*New PG = 0.50 + [linear approximation × 26]*

Using this new PG, the Kaplan-Meier curve for each study was computed, and the 95% CI of the 6-month survival was calculated with Greenwood’s formula. If the lower confidence bound of the CI was greater than the new PG, then a study was considered to have achieved success under the new end point and PG.

## Results

The study population consisted of 905 patients treated with catheter ablation for antiarrhythmic drug–refractory paroxysmal AF in the AdmIRE, Q-FFICIENCY, STELLAR, and SURPOINT studies. Patient characteristics are presented in Table 2.

**Table 2.**
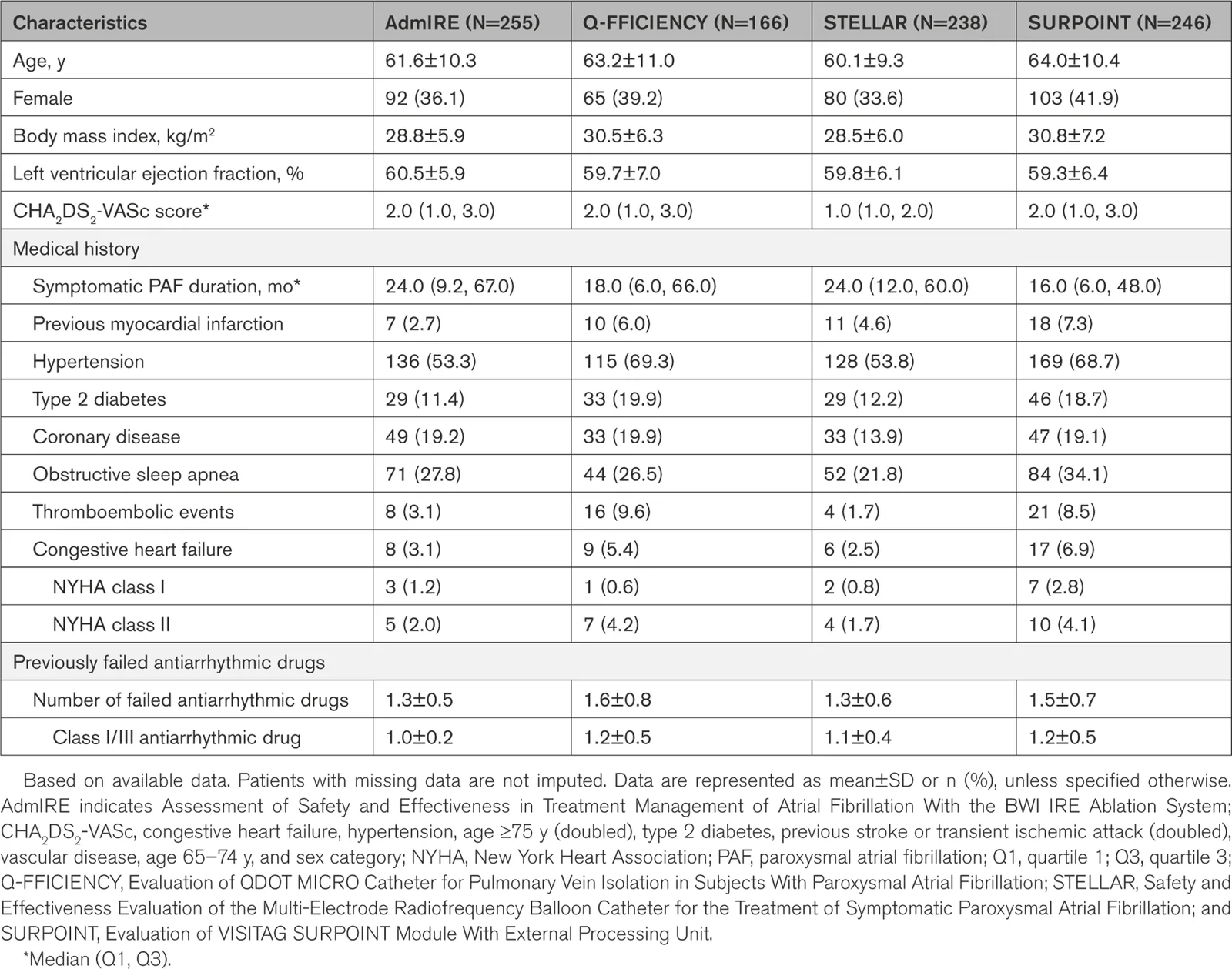
Patient Characteristics

### End Point Concordance

Table 3 displays summary statistics of the 2-by-2 tables, by study. The current and proposed effectiveness end points showed high agreement, with concordance ranging from 83% to 92%. Sensitivity rates ranged from 57% to 76%, indicating that most recurrences were detected by 6 months of follow-up. Specificity ranged from 91% to 98%. Positive predictive values ranged from 76% to 89%, and negative predictive values ranged from 86% to 93%. Both the positive predictive values and negative predictive values suggest a reasonable level of accuracy in defining procedural success with the shortened blanking period and follow-up duration.

**Table 3.**
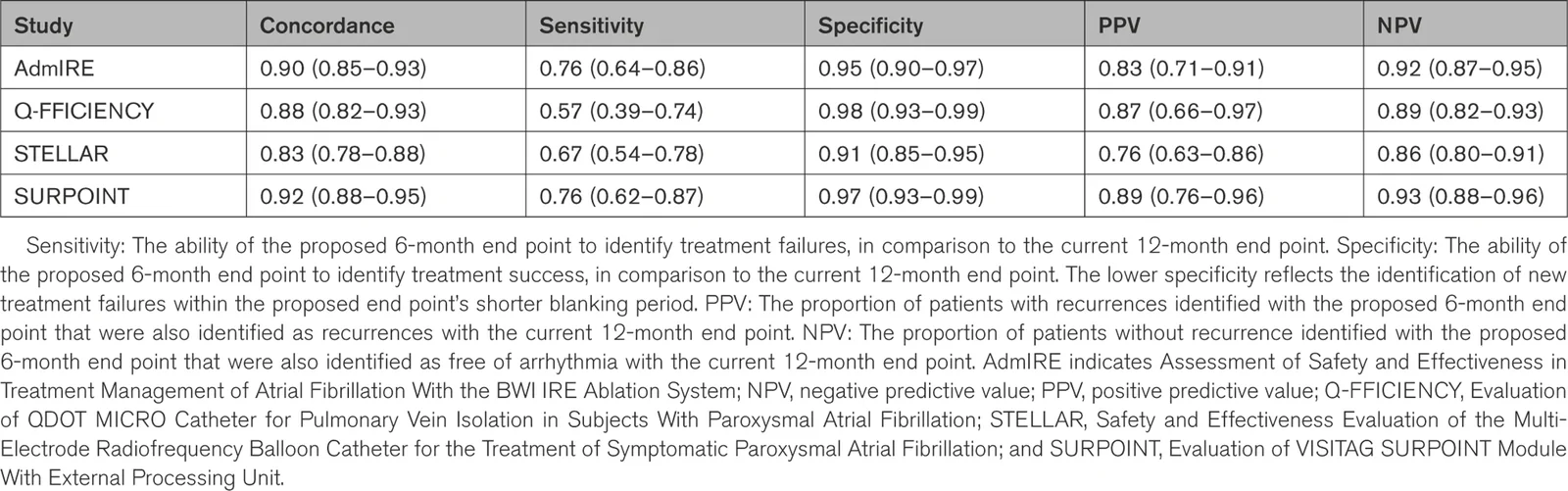
Summary Statistics of Comparisons Between the Current and Proposed Effectiveness End Points With 95% CI

### Linear Approximation Prediction

Figure displays the pooled Kaplan-Meier curve across all 4 studies, with the linear approximation superimposed from months 6 to 12. The linear approximation of the weekly change in survival probability (≈0.0024) fitted the Kaplan-Meier curve well. When using the 6-month Kaplan-Meier estimates to predict the 12-month survival for individual studies, the linear approximation provided accurate predictions for the pooled cohort (predicted 12-month survival rate of 72.5% versus actual 12-month survival rate of 71.0%; pooled error of −1.5%), as well as for the individual studies (errors between −3.6% and 0.3%). Table 4 shows the predictive accuracies for each study and the pooled cohort.

**Table 4.**
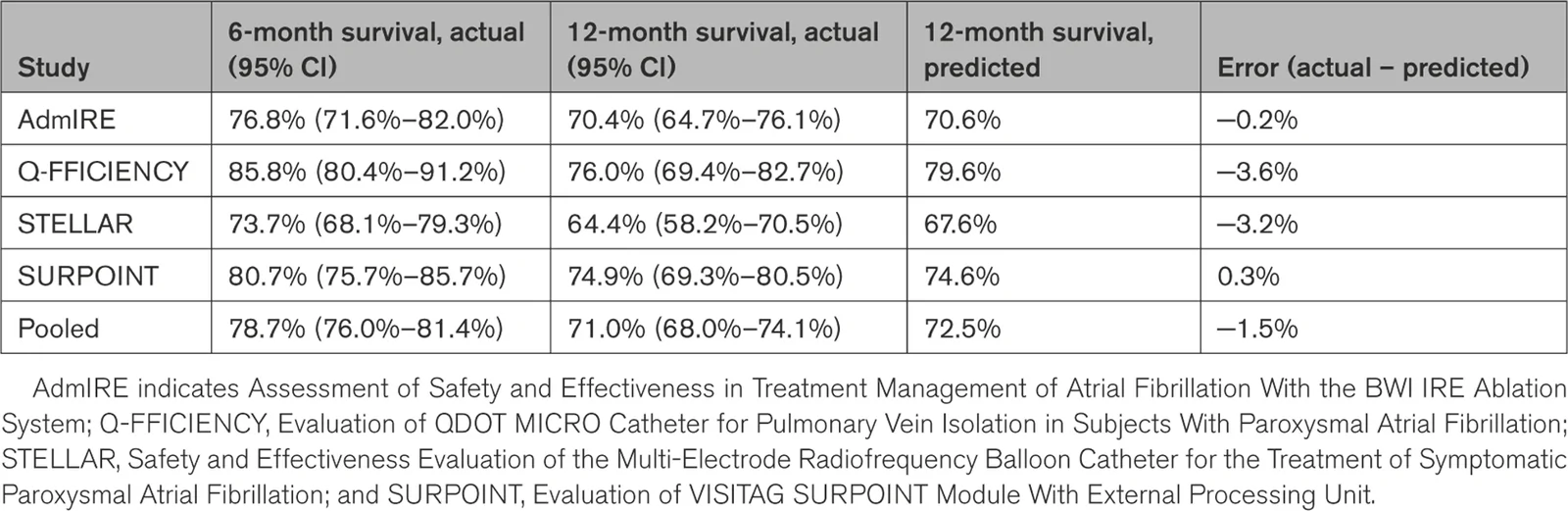
Accuracy of Linear Approximation When Using the Proposed 6-Month End Point to Predict 12-Month Results

**Figure.**
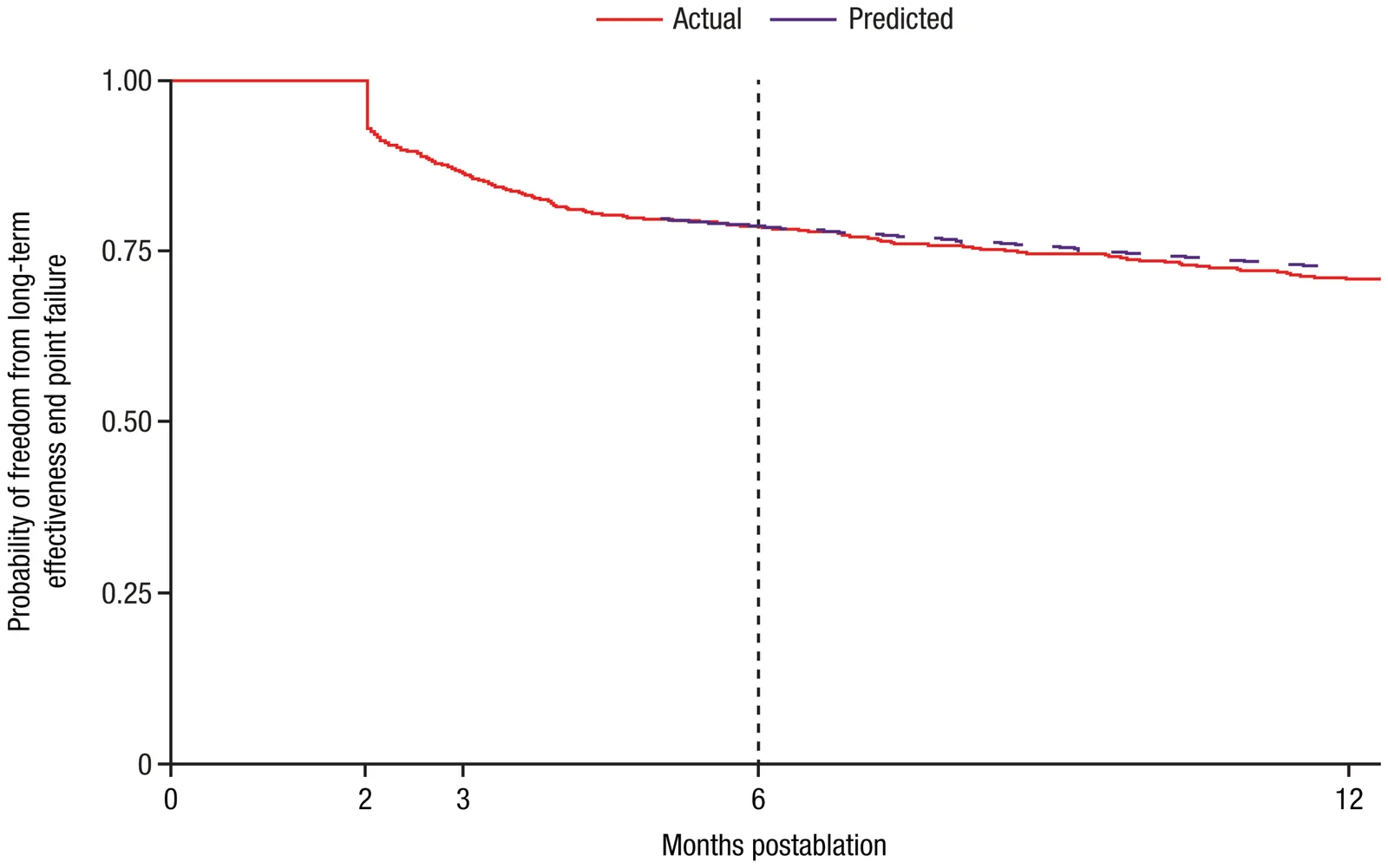
Kaplan-Meier curve with linear approximation for pooled studies.

Table S2 displays the sensitivity analysis of the linear approximation method for each study individually. The slope for the individual studies ranged from 0.0020 to 0.0029. The prediction error of the 12-month survival proportion across the 4 individual studies ranged from −4.2% to 0.7%.

Table S3 displays the results of the sensitivity analysis of the linear approximation on the pooled data with a 3-month blanking period, where the slope of the linear approximation is ≈0.0042. The prediction error across the 4 studies ranged from −0.5% to 3.3%, and the pooled error was 1.6%.

Table S4 displays the results of the sensitivity analysis on the linear approximation on the pooled data with the alternative end point definition. The accuracy is preserved, with error rates between the studies ranging from −0.3% to 3.9%, and a pooled error of 1.9%.

### New PG

Using the 12-month clinical effectiveness success rate of 50% as a benchmark, the corresponding 6-month success threshold was 56.2%, based on the pooled linear approximation of 0.0024. Applying this success criterion of 56.2% to the 6-month end point would result in all 4 studies being deemed a success for the long-term recurrence end point. Given that all 4 studies were able to claim success with their original study designs and end point definitions, this indicates the changed end point and PG would not have impacted the determination of success for any of the included studies (Table 5).

**Table 5.**
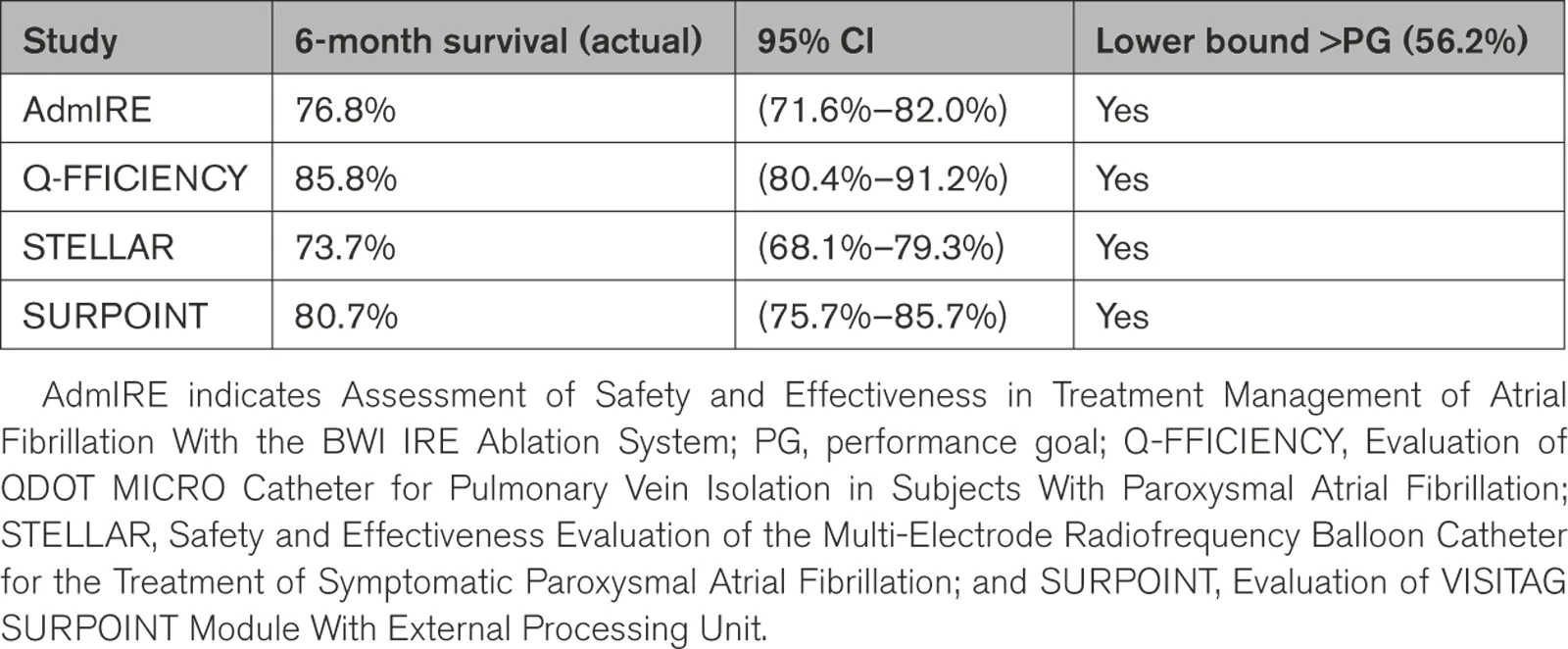
Study Success With the Proposed 6-Month End Point and 56.2% Performance Goal

## Discussion

The current study demonstrates the following key findings: (1) arrhythmia-based failure events are not uniformly distributed across the follow-up period; (2) most failure events occur within the first few months of active follow-up following the postintervention blanking period; and (3) the 1-year clinical effectiveness can be reliably extrapolated from the survival at 6 months with reasonable accuracy. Taken together, these findings demonstrate that a study design that assesses the clinical effectiveness of a therapy at a 6-month follow-up duration (inclusive of a 2-month blanking period) would have a high level of agreement with a study design that assesses the clinical effectiveness at a 12-month follow-up duration (inclusive of a 3-month blanking period). These findings have important implications for clinical trial design, routine clinical practice, and postablation care.

Arrhythmia recurrence dynamics following intervention are complex, with atrial tachyarrhythmia recurrence following rhythm control intervention being both quantitatively and qualitatively nonuniform.^7,8^ Concordant with the current results, Aguilar et al^8^ recently performed a pooled analysis of 42 clinical trials evaluating catheter ablation of paroxysmal AF (10 246 patients). Despite heterogeneous noninvasive monitoring protocols, the combined analysis demonstrated that the yield of atrial tachyarrhythmia detection is highest in the first few months of active arrhythmia monitoring and progressively decreases thereafter. While there is no follow-up duration beyond which arrhythmia will no longer recur, the nonuniform nature of arrhythmia recurrence observed in these studies suggests that increasing clinical follow-up durations is of progressively lower value, with the cost-effectiveness of rhythm monitoring decreasing as a function of time.^7,8^

For further validation, our linear approximation model was applied to the data in the Aguilar et al^8^ data set. As the 42 studies incorporated a 3-month blanking period, the linear model with the 3-month blanking period (from the sensitivity analysis, with a slope of ≈0.0042) was used for validation. The resulting prediction of 12-month success was 72.48%, which is only 0.32% off from the reported success rate of 72.8%. In effect, linear approximation resulted in a more accurate estimate of 12-month outcome compared with the prediction formula employed by Aguilar et al^8^ (prediction error of 0.32% versus 4.2%).

Moreover, recent evidence suggests that the timing of arrhythmia recurrence is of critical importance, indicating that earlier recurrences are qualitatively different from later recurrences. A combined patient-level analysis of the CIRCA-DOSE and EARLY-AF randomized clinical trials, which employed continuous cardiac rhythm monitoring through implantable devices, demonstrated that ≈80% of patients experienced a first arrhythmia recurrence within 6 months of treatment initiation (4 months active follow-up plus 2 months postprocedural blanking).^7^ Perhaps more important is the observation that those patients with recurrence within 6 months of follow-up had the greatest AF burden at 1 year postrecurrence and at 3 years of follow-up, suggesting that there is a fundamental difference in the clinical relevance and mechanism of recurrence between those with recurrences before and after 6 months of follow-up.

Taken together, these findings suggest that the use of a 12-month follow-up may not be necessary when the primary goal of a study is to detect clinically relevant recurrences of AF. Given this observation, it may be possible to substantially truncate clinical trial follow-up, resulting in substantial operational savings, earlier dissemination of results, and faster implementation of novel technologies into clinical practice.

### Limitations

There are several limitations to this analysis. First, the patients included in the current analysis had symptomatic paroxysmal AF and were referred for catheter-based intervention. We cannot specifically extrapolate the results to a population with persistent AF or those being treated with pharmacotherapy. Second, the studies included all employed similar monitoring strategies (Table 1). While the accuracy may differ with studies employing different monitoring protocols, we observed reasonable accuracy when our model results were applied to a data set of 42 trials of variable monitoring protocols evaluating 10 246 patients and published between 2014 and 2024. Still, the impact of alternate rhythm monitoring strategy on the sensitivity of arrhythmia detection with a shortened follow-up period remains to be investigated. Lastly, the linear approximation may only be suitable for limited (eg, 6-month) extrapolations of long-term effectiveness (eg, 12-month outcomes). The use of extrapolation beyond this duration has not been validated.

### Conclusions

The timing of arrhythmia recurrence after catheter ablation of paroxysmal AF is nonuniform, with the yield of active monitoring decreasing as time increases from the index intervention. Shortening the postablation follow-up period may allow for a shorter, less expensive clinical trial without significantly compromising the detection of recurrent arrhythmia after paroxysmal AF catheter ablation.

## ARTICLE INFORMATION

### Acknowledgments

The authors thank all Biosense Webster, Inc. investigators, study personnel, and patients for their valuable trial participation. The authors thank Julie Taylor, Ibukun Fowe, Lycely Sepulveda-Torres, and the Biosense Webster, Inc. clinical and biostatistics team for their efforts in trial execution, statistical analysis, and input during this article’s development. Medical writing and editorial assistance were provided in accordance with Good Publication Practice guidelines by Lumanity Communications, Inc., under the guidance of the authors, and were funded by Biosense Webster, Inc. (Irvine, CA), part of Johnson & Johnson MedTech.

### Disclosures

Dr Natale reports receiving consulting fees from Abbott, Biosense Webster, Inc., part of Johnson & Johnson MedTech, Biotronik, Boston Scientific, and iRhythm; and support for attending meetings and travel from Boston Scientific and Biosense Webster, Inc., part of Johnson & Johnson MedTech. S. Hynes and Dr Jamshidian are employees of Biosense Webster, Inc., part of Johnson & Johnson MedTech. The other author reports no conflicts.

### Supplemental Material

Tables S1–S4

## Supplementary Material
