# Supplementary material for: Incorporating a Shortened Long-Term Effectiveness End Point Into Catheter Ablation Trials: Statistical Properties and Implications
Source: Circ Arrhythm Electrophysiol. 2026 Sep 18;19(10):e014773. doi: 10.1161/CIRCEP.125.014773 (PMC13686623; doi:10.1161/CIRCEP.125.014773)
Supplement: Supplementary file 1 [file hae-19-e014773-s001.pdf]

Incorporating a Shortened Long-Term Effectiveness End Point into Catheter Ablation Trials: Statistical Properties and Implications

Jason G Andrade[1,2,3]; Stephen Hynes, MS[4]; Farid Jamshidian, PhD[4]; Andrea Natale, MD[5,6]

[1]Department of Medicine, Vancouver General Hospital, Vancouver, Canada; [2]Center for Cardiovascular Innovation, Vancouver, Canada; [3]Department of Medicine, Montréal Heart Institute, Université de Montréal, Montréal, Canada; [4]Biosense Webster Inc., part of Johnson & Johnson MedTech, Irvine, CA, USA; [5]Texas Cardiac Arrhythmia Research Foundation, Austin, TX, USA; [6]Division of Cardiology, Department of Biomedicine and Prevention, University of Tor Vergata, Rome, Italy

Supplemental Material

Table S1. Summary of Monthly Kaplan-Meier Estimates for Long-Term Effectiveness Survival, Pooled Analysis

Month	Survival at start	Survival at end	Change in survival
(0, 1)	100.00%	100.00%	0.00%
(1, 2)	100.00%	100.00%	0.00%
(2, 3)	100.00%	86.12%	13.88%
(3, 4)	86.12%	81.49%	4.62%
(4, 5)	81.49%	79.68%	1.81%
(5, 6)	79.68%	78.66%	1.02%
(6, 7)	78.66%	77.17%	1.49%
(7, 8)	77.17%	75.79%	1.38%
(8, 9)	75.79%	74.63%	1.16%
(9, 10)	74.63%	73.70%	0.93%
(10, 11)	73.70%	72.29%	1.41%
(11, 12)	72.29%	71.03%	1.26%

Table S2. Study-Level Accuracy of the Linear Approximation When Using the Proposed 6-Month End Point to Predict 12-Month Results

Study	Slope	12-Month survival, actual (95% CI)	12-Month survival, predicted	Error (actual – predicted)
AdmIRE	0.0027	70.4% (64.7%, 76.1%)	69.7%	0.7%
Q-FFICIENCY	0.0029	76.0% (69.4%, 82.7%)	78.2%	−2.2%
STELLAR	0.0020	64.4% (58.2%, 70.5%)	68.6%	−4.2%
SURPOINT	0.0020	74.9% (69.3%, 80.5%)	75.6%	−0.7%

CI, confidence interval.

Table S3. Accuracy of Linear Approximation (Pooled Analysis, 3-Month Blanking) to Predict 12-Month Results

Study	6-Month survival, actual (95% CI)	12-Month survival, actual (95% CI)	12-Month survival, predicted	Error (actual – predicted)
AdmIRE	82.2% (77.5%, 86.9%)	74.6% (69.2%, 80.1%)	71.3%	3.3%
Q-FFICIENCY	88.2% (83.2%, 93.2%)	77.8% (71.3%, 84.3%)	77.3%	0.5%
STELLAR	82.6% (77.8%, 87.5%)	71.2% (65.3%, 77.2%)	71.7%	−0.5%
SURPOINT	85.2% (80.7%, 89.7%)	76.8% (71.3%, 82.2%)	74.3%	2.5%
Pooled	84.2% (81.8%, 86.6%)	74.9% (72.0%, 77.8%)	73.3%	1.6%

CI, confidence interval.

Table S4. Accuracy of Linear Approximation (Long-Term Effectiveness End Point with Alternative Failure Modes, Pooled Analysis, 2-Month Blanking) to Predict 12-Month Results

Study	6-Month survival, actual (95% CI)	12-Month survival, actual (95% CI)	12-Month survival, predicted	Error (actual – predicted)
AdmIRE	78.3%<br>(73.3%, 83.4%)	72.0%<br>(66.4%, 77.6%)	68.1%	3.9%
Q-FFICIENCY	88.2%<br>(83.2%, 93.2%)	78.4%<br>(72.0%, 84.8%)	78.0%	0.4%
STELLAR	80.9%<br>(75.9%, 85.9%)	70.4%<br>(64.5%, 76.4%)	70.7%	−0.3%
SURPOINT	87.3%<br>(83.1%, 91.6%)	79.8%<br>(74.6%, 85.0%)	77.1%	2.7%
Pooled	83.2%<br>(80.8%, 85.7%)	74.9%<br>(72.0%, 77.8%)	73.0%	1.9%

CI, confidence interval.
